# Remodeling of macular vortex veins in pachychoroid neovasculopathy

**DOI:** 10.1038/s41598-019-51268-9

**Published:** 2019-10-11

**Authors:** Hidetaka Matsumoto, Shoji Kishi, Ryo Mukai, Hideo Akiyama

**Affiliations:** Department of Ophthalmology, Gunma University, School of Medicine, Maebashi, Japan

**Keywords:** Macular degeneration, Diagnostic markers

## Abstract

Superior and inferior macular vortex veins are divided by a horizontal watershed passing through the macula. We evaluated macular vortex vein remodeling in eyes with pachychoroid neovasculopathy (PNV). Thirty eyes of 30 patients with treatment-naïve PNV and 30 normal eyes of 30 age-, gender-, and refraction-matched subjects were studied. We assessed the features of macular vortex veins employing en face optical coherence tomography (OCT) and determined central choroidal thickness (CCT) using B-mode OCT. Of the 30 normal eyes, a horizontal watershed was identified in 24 eyes (80%), while venous anastomosis between the superior and inferior vortex veins was observed in 6 eyes (20%). Mean CCT was 233 μm. Of the 30 eyes with PNV, vortex veins were dilated in all 30 eyes with PNV. In 27 of the 30 PNV eyes (90%), the horizontal watershed had disappeared, and collateral veins had instead developed via anastomosis between the superior and inferior vortex veins, making this finding significantly more frequent than in normal eyes (*P* < 0.001). Mean CCT was 357 μm, significantly thicker than that of normal eyes (*P* < 0.001). Remodeling of choroidal drainage routes by venous anastomosis between superior and inferior vortex veins was common in eyes with PNV. This observation suggests longstanding congestion of the choroidal veins.

## Introduction

Pachychoroid neovasculopathy (PNV) is a macular disorder, first proposed in 2015 by Pang and Freund, which shows type 1 choroidal neovascularization (CNV) accompanied by pachychoroid in the absence of characteristic features of neovascular age-related macular degeneration (AMD)^1^. The term “pachychoroid” refers to an abnormal increase in choroidal thickness often associated with dilated choroidal vessels^2^. PNV is one of the pachychoroid spectrum diseases, which include central serous chorioretinopathy (CSC) and polypoidal choroidal vasculopathy (PCV)^3,4^. CSC can reportedly progress to PNV or PCV with the development of type 1 CNV^5–7^.

The vortex veins serve as choroidal drainage routes passing through the sclera. Horizontal and vertical watersheds divide the choroidal vasculature into four quadrants. In the temporal hemisphere of the eye, superior and inferior vortex veins independently serve the two corresponding quadrants^8^. Our recent study using en face optical coherence tomography (OCT) revealed asymmetry of the superior and inferior vortex veins to be common in CSC, as well as marked dilation of the dominant vortex veins^9^. Using ultra-widefield indocyanine green angiography (ICGA), Pang and colleagues showed involvement of the dominant vortex veins in the entire posterior pole and that these veins were dilated from the distal end to the ampulla in eyes with CSC^10^. Dansingani *et al*. described dilated outer choroidal vessels, so-called “pachyvessels”, in pachychoroid spectrum diseases^11^. Thus, we can reasonably speculate that vortex veins might become congested in pachychoroid spectrum diseases, thereby leading to dilated outer choroidal vessels.

Experimental studies and clinical cases with vortex vein occlusion have shown choroidal veins to undergo marked collateral formation which compensates for choroidal venous congestion^12–15^. We recently used en face and B-mode OCT images of the choroid to study CSC^9,16^. Superior and inferior vortex veins were commonly seen as being asymmetric in acute CSC. However, such asymmetry was less evident in chronic CSC due to collateral vessels between the superior and inferior vortex veins. Moreover, the measured central choroidal thickness (CCT) value was significantly greater in acute than in chronic CSC. Collateral vessels may compensate for the choroidal congestion, which would in turn lead to decreased choroidal thickness. Herein, we investigated the remodeling of macular vortex veins in eyes with PNV.

## Results

The demographic and clinical characteristics of our PNV patients and normal subjects are presented in Table 1. The normal subjects included 20 men (66.7%) and 10 women (33.3%). The age of the normal subjects was 63.1 ± 9.2 years (mean ± standard deviation). All eyes were phakic with a refraction of −0.47 ± 2.17 diopters. The PNV patients in this study included 26 men (86.7%) and four women (13.3%). Their age was 64.1 ± 11.6 years. Twenty-six eyes (86.7%) were phakic and four eyes (13.3%) had pseudophakia. The refraction of the 26 phakic eyes was −0.21 ± 1.79 diopters. Best corrected visual acuity was 0.23 ± 0.28 logMAR units.

**Table 1:** Demographic and clinical characteristics of normal subjects and PNV patients.

	Normal	PNV	P Value
Number of eyes	30	30	
Age (years)	63.1 ± 9.2	64.1 ± 11.6	0.662
Male/female	20/10	26/4	0.067
Refraction (diopters)	−0.47 ± 2.17	−0.21 ± 1.79	0.323
Symmetry/asymmetry of vortex veins	19/11	15/15	0.297
Vortex vein anastomosis +/−	6/24	27/3	<0.001
Central choroidal thickness (µm)	233 ± 77	357 ± 100	<0.001
Dilated vortex veins under CNV	—	30	

PNV = pachychoroid neovasculopathy; CNV = choroidal neovascularization.

**Figure 1:**
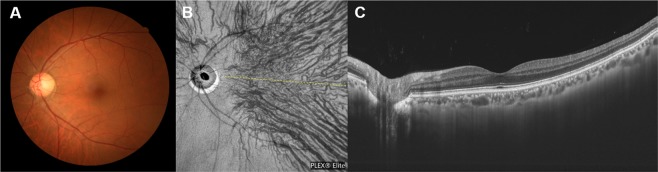
Images of a normal eye of a 56-year-old man. The refraction in the left eye was +0.50 diopters. (**A**) Color fundus photograph shows a normal fundus appearance. (**B**) En face optical coherence tomography (OCT) image (12 mm × 12 mm) showing vortex veins in the deep layer of the choroid. Superior and inferior vortex veins are symmetrical and there is a horizontal watershed zone (dashed line). (**C**) 12 mm horizontal B-mode OCT image through the fovea shows normal appearances of the retina and choroid. The central choroidal thickness is 194 µm.

Of the 30 normal eyes, superior and inferior vortex veins were symmetrically distributed in 19 (63.3%) (Fig. 1), while being asymmetric in 11eyes (36.7%), 6 eyes (20%) with superior dominant and 5 eyes (16.7%) with inferior dominant findings. We identified a horizontal watershed in 24 eyes (80%) (Fig. 1). Anastomoses between the superior and inferior vortex veins were present in 6 eyes (20%), in which the watershed zone between the superior and inferior vortex veins was not identified. CCT was 233 ± 77 µm.

**Figure 2:**
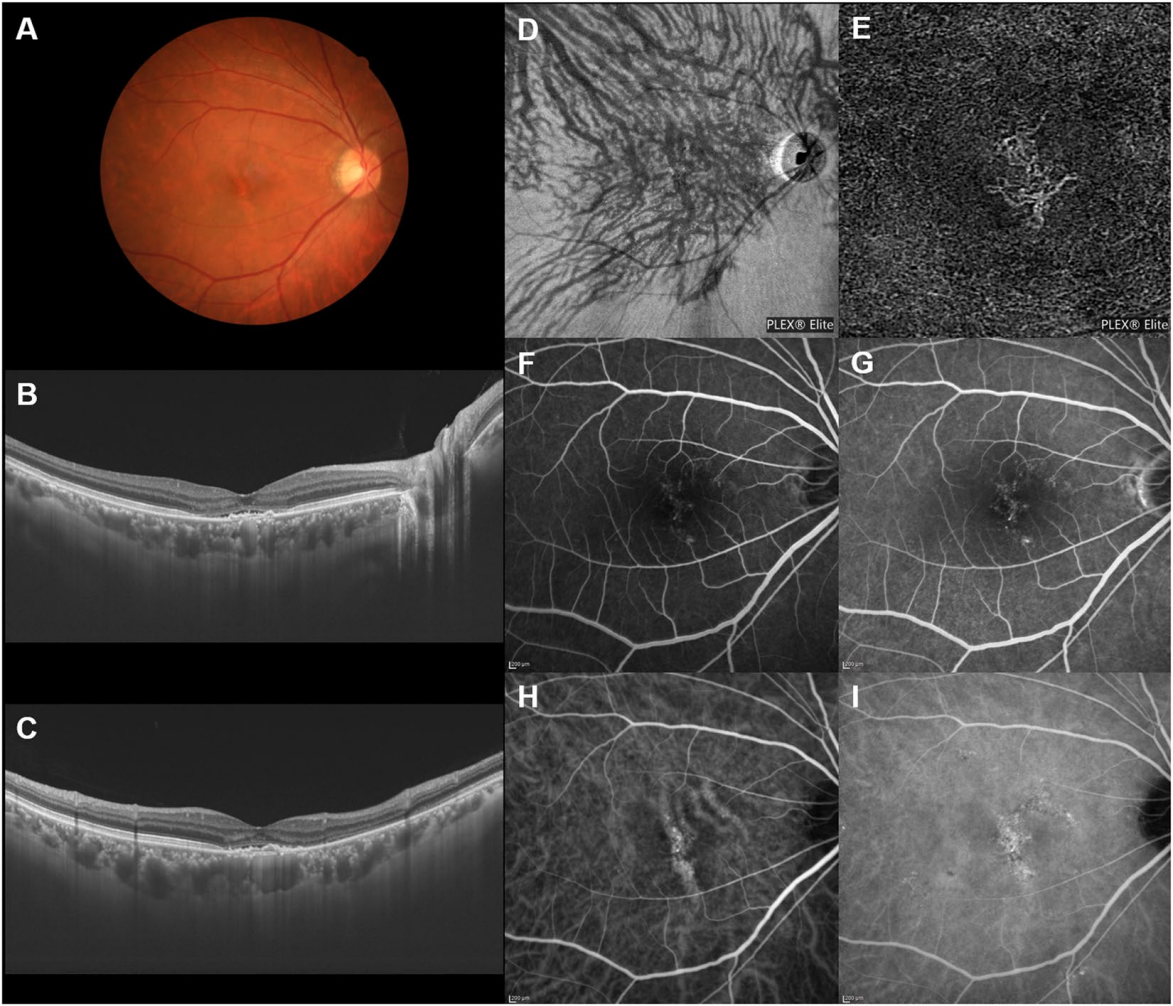
Images of an eye with pachychoroid neovasculopathy in a 53-year-old man. The refraction in the right eye was +0.00 diopters. Best-corrected visual acuity in the right eye was −0.08 logarithm of the minimum angle of resolution unit. (**A**) Color fundus photograph shows a retinal pigment epithelium (RPE) abnormality at the fovea. A large choroidal vessel can be seen under the fovea. (**B**,**C**) 12 mm horizontal and vertical B-mode optical coherence tomography (OCT) images through the fovea show pachychoroid with dilated outer choroidal vessels (vortex veins). A shallow irregular RPE detachment accompanied by slight serous retinal detachment is observed at the fovea. The central choroidal thickness is 353 µm. (**D**) En face OCT image (12 mm × 12 mm) showing dilated vortex veins in the deep layer of the choroid. Superior vortex veins are dominant as compared to the inferior vortex veins. The horizontal watershed zone has disappeared, showing instead collateral veins due to anastomoses between the superior and inferior vortex veins. (**E**) OCT angiography (3 mm × 3 mm) shows network vessels of choroidal neovascularization (CNV) between the detached RPE and Bruch’s membrane. CNV was detected over the dilated vortex veins. (**F**,**G**) Fluorescein angiography (early and late phases) shows window defects and some oozing at the fovea. (**H**,**I**) Indocyanine green angiography (early and late phases) shows dilated choroidal vessels with hyperpermeability at the macular area and suspected CNV at the fovea.

**Figure 3:**
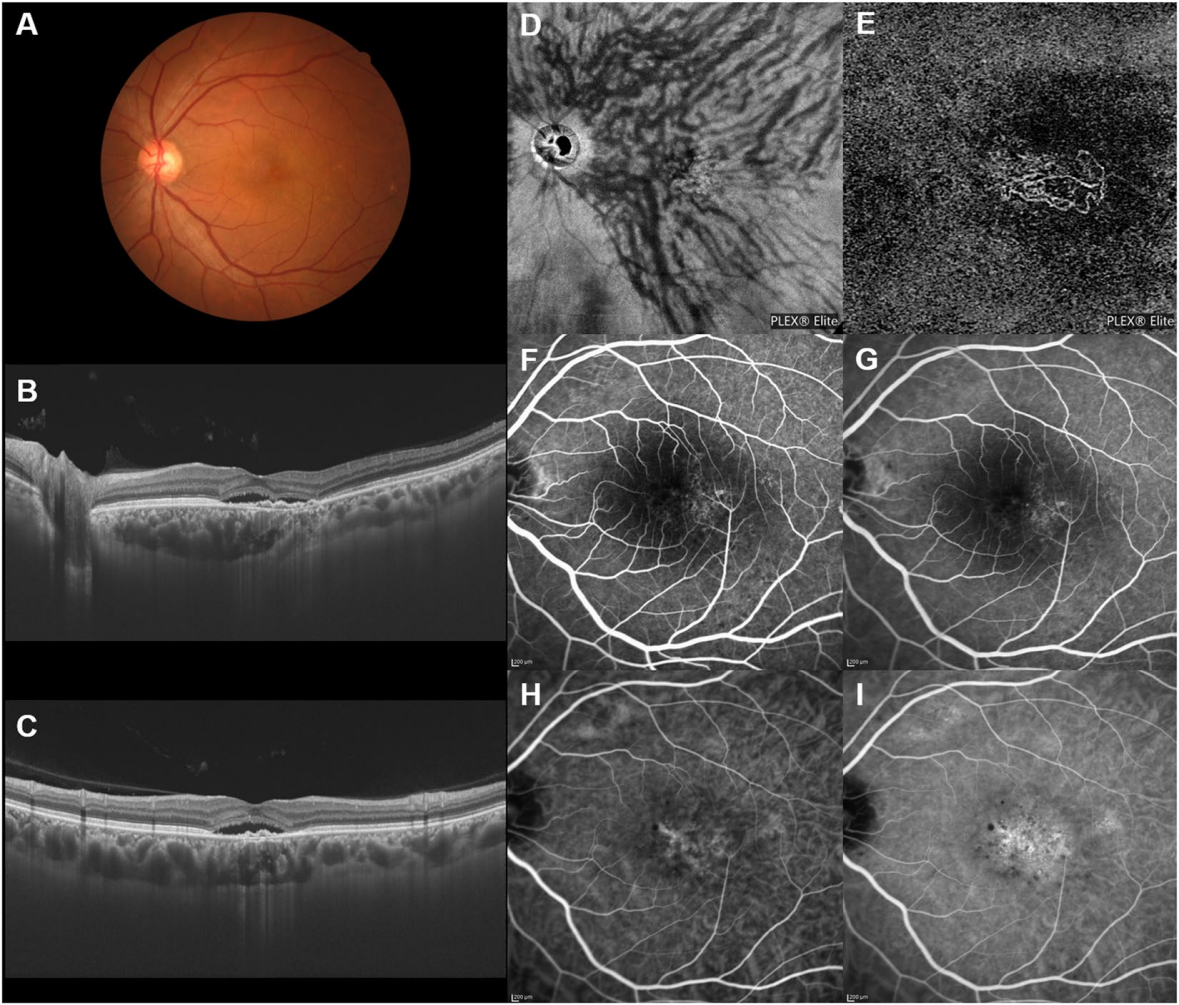
Images of an eye with pachychoroid neovasculopathy in a 54-year-old man. The refraction in the left eye was −1.00 diopter. Best-corrected visual acuity in the left eye was 0.22 logarithm of the minimum angle of resolution unit. (**A**) Color fundus photograph shows a retinal pigment epithelium (RPE) abnormality at the macular area. (**B**,**C**) 12 mm horizontal and vertical B-mode OCT images through the fovea show pachychoroid with dilated outer choroidal vessels (vortex veins). Dilated vortex veins are notable between the papilla and subfovea in the horizontal B-mode OCT image. A shallow irregular RPE detachment accompanied by serous retinal detachment is observed at the fovea. The central choroidal thickness is 386 µm. (**D**) En face OCT image (12 mm × 12 mm) showing dilated vortex veins in the deep layer of the choroid. Superior and inferior vortex veins are symmetrical. The horizontal watershed zone has disappeared, with collateral veins due to anastomoses between the superior and inferior vortex veins instead being observed in the peripapillary area. (**E**) OCT angiography (3 mm × 3 mm) shows network vessels of choroidal neovascularization (CNV) between the detached RPE and Bruch’s membrane. CNV was detected over the dilated vortex veins. (**F**,**G**) Fluorescein angiography (early and late phases) shows window defects and some oozing at the macular area. (**H**,**I**) Indocyanine green angiography (early and late phases) shows suspected CNV at the fovea and choroidal vascular hyperpermeability around the fovea.

**Figure 4:**
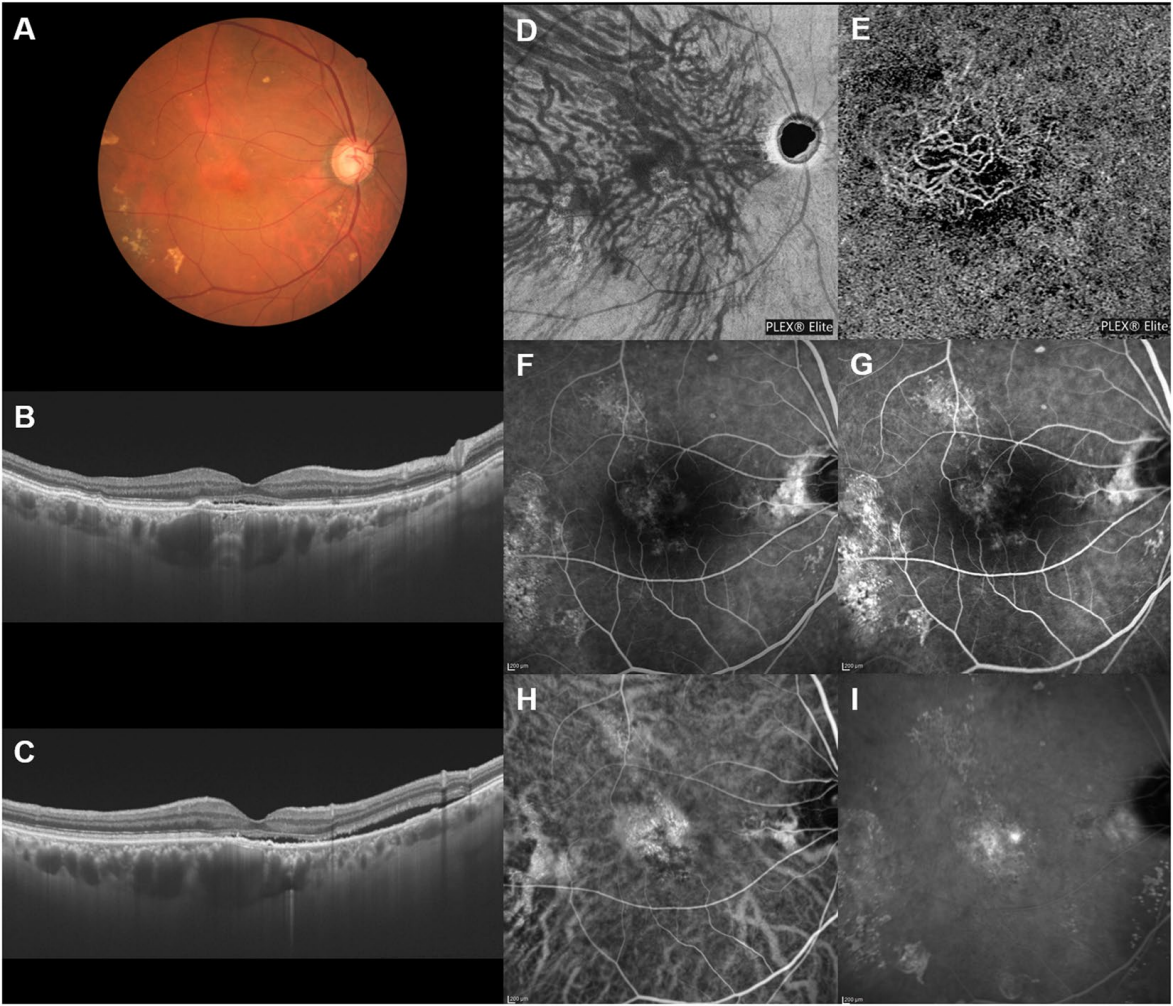
Images of an eye with pachychoroid neovasculopathy in a 68-year-old man. The refraction in the left eye was +1.50 diopters. Best-corrected visual acuity in the left eye was 0.10 logarithm of the minimum angle of resolution unit. (**A**) Color fundus photograph shows a retinal pigment epithelium (RPE) abnormality with pachydrusen around the macular area. Markedly dilated vortex veins are seen at the fovea. (**B**,**C**) 12 mm horizontal and vertical B-mode OCT images through the fovea show pachychoroid with dilated outer choroidal vessels (vortex veins). Dilated vortex veins are notable under the fovea. A shallow irregular RPE detachment accompanied by serous retinal detachment is observed at the fovea. The central choroidal thickness is 465 µm. (**D**) En face OCT image (12 mm × 12 mm) showing dilated vortex veins in the deep layer of the choroid. The horizontal watershed zone has disappeared, with anastomoses between the superior and inferior vortex veins instead being observed. The collateral veins at the macula show sinusoid like dilatation. (**E**) OCT angiography (3 mm × 3 mm) shows network vessels of choroidal neovascularization (CNV) between the detached RPE and Bruch’s membrane. CNV was detected over the dilated vortex veins. (**F**,**G**) Fluorescein angiography (early and late phases) shows window defects at the posterior pole of the fundus and some oozing in the macular area. (**H**,**I**) Indocyanine green angiography (early and late phases) shows suspected CNV at the fovea, choroidal vascular hyperpermeability and punctate hyperfluorescent spots corresponding to pachydrusen around the macular area.

The en face OCT showed dilated vortex veins (outer choroidal vessels) in all 30 PNV eyes. Fifteen eyes (50%) showed a symmetrical distribution of the superior and inferior vortex veins. The superior and inferior vortex veins were both dilated in the posterior fundus. The other 15 eyes (50%) showed an asymmetrical vortex vein distribution, 8 eyes (26.7%) with superior dominant and 7 eyes (23.3%) with inferior dominant findings. Dominant vortex veins showed somewhat greater dilatation. Twenty-seven eyes (90%) showed anastomosis between the superior and inferior vortex veins at the horizontal watershed (Figs 2–4), making this finding significantly more frequent than in normal eyes (*P* < 0.001). In 6 of the 30 PNV eyes (20%), the anastomosis was observed mainly in the peripapillary area (Fig. 3). The vortex vein anastomosis showed sinusoid like dilatation in 2 eyes (6.7%) (Fig. 4). CCT was 357 ± 100 µm, significantly thicker than that of normal eyes (*P* < 0.001). ICGA confirmed perfusion in the collateral vessels which had developed from anastomosis. OCT angiography demonstrated network vessels of CNV between the detached retinal pigment epithelium (RPE) and Bruch’s membrane, which consistently arose at the site of dilated vortex veins in all 30 eyes with PNV (Figs 2–4).

## Discussion

We conducted a retrospective investigation of 30 eyes with treatment-naïve PNV using multimodal imaging. En face OCT revealed an anastomosis between the superior and inferior vortex veins in 27 eyes (90%) with PNV, from which the horizontal watershed zone had disappeared (Figs 2–4). Sub-RPE CNV had consistently arisen at the site of dilated vortex veins in all 30 PNV eyes. In 24 of the 30 normal eyes (80%), as no anastomosis was present, the horizontal watershed zone was identified between the superior and inferior vortex veins (Fig. 1).

Collateral formation due to venous anastomosis at the watershed appears to be a common response to choroidal vortex vein congestion. Takahashi and Kishi evaluated choroidal drainage routes by applying wide-angle ICGA in eyes with occluded vortex veins after scleral buckling surgery for retinal detachment^15^. In 10 of the 12 eyes that underwent angiography 3 months or more postoperatively, the collateral veins that developed connected the sector harboring the occluded vortex veins to that with intact vortex veins via venovenous anastomoses^15^. The collateral veins which compensate for the occlusion of vortex veins also formed as a consequence of radiation retinochoroidopathy^14^. Hayreh and Baines devised an experimental model of vortex vein occlusion based on cauterization of vortex veins in monkey eyes^12^. Fluorescein angiography in the model eye showed delayed choroidal filling in the sector the occluded vortex vein in the acute stage, though the filling normalized in approximately 1 or 2 weeks^12^. Okada and colleagues developed another experimental vortex vein occlusion model based on ligating the vortex veins in rabbit eyes^13^. Their plastic cast study demonstrated collateral routes between the areas of occluded and intact vortex veins^13^. These reported results indicate the considerable plasticity of choroidal veins, which allows remodeling of drainage routes to compensate for choroidal venous congestion. We observed anastomosis between superior and inferior vortex veins in 27 of 30 eyes (90%) with PNV in this study (Figs 2–4). Longstanding congestion of vortex veins might lead to the remodeling of choroidal drainage routes by collateral vessels connecting the superior and inferior vortex veins in eyes with PNV.

A recent study focusing on the choroidal features of peripapillary PCV found that eyes with this type of PCV had thinner subfoveal choroids than those with macular PCV^17^. However, at the foci of extrafoveal disease, choroidal thickness, Haller’s layer thickness, and its ratio to total choroidal thickness were all relatively high, findings consistent with those of macular PCV^17^. Peripapillary pachychoroid syndrome is a newly proposed variant of pachychoroid disease spectrum, which demonstrates thicker nasal versus temporal macular choroidal layers^18^. Peripapillary choroidal thickening is reportedly associated with nasal macular intraretinal and/or subretinal fluid accumulation and also with disk edema in some cases^18^. In our present study, 6 PNV cases (20%) showed anastomosis between the superior and inferior vortex veins mainly in the peripapillary area. Peripapillary anastomosis between the superior and inferior vortex veins might develop in peripapillary PCV as well as in peripapillary pachychoroid syndrome. The peripapillary watershed zone appears to be another preferential site, besides the macula, of anastomosis development between the superior and inferior vortex veins.

Lee and colleagues described attenuation and thinning of the choriocapillaris and Sattler vessels overlying dilated outer choroidal vessels (pachyvessels), based on their investigation evaluating B-mode OCT features in eyes with PCV^4^. They suggested the following mechanism to underlie the development of CNV in eyes with PCV: Attenuation of the choriocapillaris produces a relatively ischemic environment at the RPE-Bruch membrane complex level, triggering expressions of various angiogenic factors^4^. We previously reported a choriocapillaris filling delay in CSC in the area of dilated vortex veins. In the current study, dilated vortex veins were consistently observed beneath the CNV in eyes with PNV. Therefore, outer choroidal vessel dilatation associated with chronic choriocapillaris ischemia might lead to the development of CNV in eyes with PNV.

The limitations of our study include its retrospective nature, the single-center design, and the relatively small number of patients. OCT focused on the posterior pole of the fundus demonstrates only the posterior portion of the choroidal circulation. Measurements of CCT were performed manually. The current subjects were all Japanese, such that the results may not be generalizable to a larger PNV population, including other racial groups.

In conclusion, remodeling of choroidal drainage routes via venous anastomosis between the superior and inferior vortex veins commonly develops in eyes with PNV, suggesting longstanding congestion of the choroidal veins.

## Methods

We performed this study, in compliance with the Declaration of Helsinki guidelines, after obtaining approval from the Institutional Review Board of Gunma University Hospital. Informed consent was obtained from all individual participants included in the present study.

### Subjects

We retrospectively studied 30 eyes of 30 patients with previously untreated PNV and 30 normal eyes of 30 age-, gender-, and refraction-matched subjects, followed clinically from April 2017 through October 2018 at Gunma University Hospital.

### Multimodal imaging methods

All patients with PNV underwent a complete ophthalmological examination, including slit-lamp biomicroscopy with a noncontact fundus lens (SuperField lens; Volk Optical Inc., Mentor, OH), color fundus photography and fundus autofluorescence (FAF) (Canon CX-1; Canon, Tokyo, Japan), FA and ICGA with an angle of 30 degrees (Spectralis HRA + OCT; Heidelberg Engineering, Heidelberg, Germany), and swept-source OCT (SS-OCT) (DRI OCT-1 Triton; Topcon Corp, Tokyo, Japan, and PLEX Elite 9000; Carl Zeiss Meditec, Dublin, CA, USA).

The DRI OCT-1 Triton and PLEX Elite 9000 devices incorporate a tunable laser with a 1050 nm central wavelength and acquire 100,000 A-scans/second. The DRI OCT-1 Triton has an axial resolution of 8 μm and a lateral resolution of 20 μm, while the PLEX Elite 9000 has an axial resolution of 6.5 μm and a lateral resolution of 20 μm. We obtained B-mode images of the horizontal and vertical line scans (12 mm) through the fovea employing the DRI OCT-1 Triton. Next, cube data were obtained with a raster scan protocol of 1024 (horizontal) × 1024 (vertical) B-scans, which covered the 12 × 12 mm area centered on the fovea by the PLEX Elite 9000. En face images were obtained from the vitreous to the choroidoscleral border with coronal slices from a 3-dimensional dataset included in the inner software. Then, we performed OCT angiography volume scanning, i.e. 300 × 300 pixels in the 3 × 3 mm area demonstrated by the PLEX Elite 9000. OCT angiography is based on optical microangiography algorithm.

All normal eyes underwent color fundus photography and SS-OCT to obtain B-mode and en face structural images.

### Definition of pachychoroid neovasculopathy

Herein, we diagnosed PNV if all of the following criteria were met. (1) CNV was diagnosed or suspected by FA and ICGA in the affected eye. (2) A shallow irregular RPE detachment at the site of CNV was observed on OCT B-mode images, while network vessels of CNV were detected between the detached RPE and Bruch’s membrane on OCT angiography^19^. (3) Clinical and anatomical features of the pachychoroid phenotype were present, i.e. pathologically dilated outer choroidal vessels on en face OCT images and regional choroidal vascular hyperpermeability on ICGA images. CCT was not included among the criteria for the pachychoroid phenotype because CCT is impacted both by age and refractive errors^20^. Furthermore, eyes with normal CCT can exhibit extrafoveal choroidal thickening at sites of CNV^4^. The absence of drusen was also excluded from among the criteria applied for diagnosing the pachychoroid phenotype because pachychoroid-associated drusen, so-called pachydrusen, can be seen in pachychoroid spectrum diseases^21,22^. CNV with polypoidal lesions, so-called polypoidal choroidal vasculopathy, was excluded from the PNV category.

### Image analysis

We evaluated the remodeling of macular vortex veins; the presence of anastomosis between superior and inferior vortex veins in eyes with PNV was demonstrated using en face OCT and ICGA. The presence of dilated vortex veins, choroidal vascular hyperpermeability, and anatomical and functional anastomoses between the superior and inferior vortex veins were judged by two experienced retinal specialists (H. Matsumoto and S. Kishi), working independently of each other. CCT was measured on B-mode images using the computer-based caliper measurement tool included in the OCT system. CCT was defined as the distance between Bruch’s membrane and the margin of the choroid and sclera under the fovea.

### Statistical analysis

For statistical analyses, the Mann–Whitney U test was used to compare unpaired values of age, refraction, and CCT. The chi-squared independence test was used to determine differences in gender, vortex vein symmetry, and the incidence of vortex vein anastomosis. The data analyses were performed using Excel 2016 (Microsoft, Redmond, WA, USA) with add-in software Statcel4^23^. A P < 0.05 was considered to indicate a statistically significant difference.
